# Short versus long treatment duration for streptococcal bloodstream infection

**DOI:** 10.1017/ash.2025.29

**Published:** 2025-02-17

**Authors:** Julie Gray, Chong Zhang, Ali Earl, Emily S. Spivak

**Affiliations:** 1 Department of Pharmacy, Yale New Haven Health, New Haven, CT, USA; 2 Division of Epidemiology, Department of Internal Medicine, University of Utah Health and School of Medicine, Salt Lake City, UT, USA; 3 Department of Pharmacy, University of Utah Health, Salt Lake City, UT, USA; 4 Division of Infectious Diseases, University of Utah School of Medicine, Salt Lake City, Utah, USA

## Abstract

**Background:**

Emerging data surrounding the rise in antimicrobial resistance have prompted a shift towards shorter antibiotic durations. Studies show similar clinical outcomes comparing shorter antibiotic courses to longer ones for uncomplicated Gram-negative bloodstream infections (BSI). However, there is a lack of data to inform durations of therapy for Streptococcal BSI.

**Methods:**

This was a retrospective cohort study of patients admitted to University of Utah Health with uncomplicated Streptococcal BSI. Inverse probability of treatment weighting (IPTW) was used to estimate the average treatment effects (ATE) of antibiotics administered for 10 days or fewer (short duration) versus more than 10 days (long duration). The primary outcome was a composite of recurrent BSI, all-cause mortality, and readmissions at 30 days from end of therapy.

**Results:**

Five hundred patients were screened and 196 were included in the final analysis. The most common sources were skin and soft tissue infections. The median duration in the short and long groups were 8 (IQR, 7–10) and 15 days (IQR, 14–17), respectively. The ATE of short versus long duration of antibiotics was not significant for the composite primary outcome (18% vs 18%; OR = 1.42 [95% CI: 0.57 to 3.53]).

**Conclusions:**

We found no appreciable difference in outcomes between patients treated with short versus long antibiotic durations for uncomplicated Streptococcal BSI. Given low absolute rates of mortality and recurrent BSI, along with the lack of evidence indicating a significant difference related to treatment duration, it is reasonable to consider shorter durations. Future research is needed to confirm our findings.

## Introduction

With rising antibiotic resistance rates and a growing recognition of other potential harms related to antimicrobial use including antibiotic-related adverse events, *Clostridioides difficile* infection, and increased strain on healthcare resources, a body of literature has grown to suggest shorter antibiotic durations are as effective, and potentially safer, than longer antibiotic durations for many infectious diseases.^1,2^ Specifically, several recent randomized clinical trials show similar outcomes for patients treated with shorter antibiotic durations as compared to longer durations for uncomplicated Gram-negative bloodstream infections (BSI).^3,4^ This evidence has informed Antimicrobial Stewardship efforts to promote judicious and safe antimicrobial use in the form of shorter antibiotic durations.^5,6^

However, there are limited data surrounding the management of Streptococcal BSI. Streptococcal BSI commonly occurs as a complication of infection at a primary site, such as skin and soft tissue infections, catheter-related BSI, pneumonia, or dental infections.^7^ Invasive Group A *Streptococcus* (GAS) infections including BSI are estimated with an incidence ranging from 1.0 – 10 per 100,000 person-years.^8^ Despite the incidence and potentially high case fatality rate, little data exists to inform treatment durations for Streptococcal BSI, nor do national guidelines address recommended durations for Streptococcal infections complicated by bacteremia.^9,10^

Analyses of treatment durations for Streptococcal BSI are limited to two published retrospective studies and one abstract; all suggesting shorter durations of therapy for uncomplicated Streptococcal BSI result in similar clinical outcomes to longer treatment durations.^11–13^ In addition to there being few published evaluations of this question, there are limitations to the existing data such as inclusion of patients with identified complicated disease states that may require longer durations of therapy by nature of complexity.^11^

Uncomplicated Streptococcal BSI^14^ is generally treated with at least 14 days of antimicrobial therapy in the United States;^15^ however, there are limited published data to support this common practice. Therefore, to inform local and national practice, the purpose of this study was to compare treatment outcomes for patients receiving short versus long antibiotic treatment durations for uncomplicated Streptococcal BSI.

## Methods

### Study design and approval

This was a retrospective cohort study of all unique, consecutive adult patients admitted to University of Utah Health with uncomplicated Streptococcal BSI. Patients were identified for chart review from the University of Utah Health Enterprise Data Warehouse with admissions from January 1, 2018 to July 15, 2023 with at least one positive blood culture with a Streptococcal species. Inclusion was limited to the first eligible encounter for patients with more than one encounter meeting criteria for inclusion during the study period. The first positive blood culture for the encounter was defined as the index blood culture. Patients were included if they were at least 18 years of age, diagnosed with uncomplicated Streptococcal BSI, and received in vitro active antibiotics for at least 72 hours from the calendar date of the index blood culture. Patients were excluded if they received antibiotics for more than 21 consecutive days to assist in excluding those with complicated BSI, were diagnosed with co-infections with non-Streptococcal organisms requiring antibiotic treatment, had polymicrobial BSI, or died or were transitioned to hospice during antibiotic therapy. Uncomplicated Streptococcal BSI was defined as negative follow-up blood cultures (if collection deemed necessary by the treating clinician), clinical resolution by day 3 (defined as no fever [<38°C],] hemodynamic stability, and a trend towards a normal WBC within 72 hours of starting active antibiotic therapy), no evidence of metastatic foci, source control (if indicated), and absence of complicated infection, including endocarditis, bone and joint infections, and/or central nervous system infections. Source control was defined as the removal of any infected hardware, catheters, or devices and drainage procedure was performed of infected fluid collections, as well as imaging assurance, as needed, of no residual or metastatic sites of infection. The inclusion and exclusion criteria were confirmed by manual chart review. Patients were assigned to either the short treatment group or the long treatment group based on the number of consecutive days they received in vitro active antibiotics (including inpatient and discharge antibiotics) for 10 days or fewer (short duration) or more than 10 days (long duration).

Patient consent was not required for this retrospective chart review study. The University of Utah Institutional Review reviewed this study and deemed exempt from oversight.

### Outcomes

The primary outcome was a composite of incidence of recurrent BSI, all-cause mortality, and unplanned readmissions at 30 days from the end of antibiotic treatment (EOT). Recurrent BSI was defined as at least one positive blood culture with the same Streptococcal species collected between 72 hours after the index blood culture and the end of the 30 or 90 day follow up period. Secondary outcomes included hospital length of stay, 90-day all-cause mortality from EOT, 90-day recurrent BSI from EOT, 90-day unplanned readmissions from EOT.

### Data collection

Data were collected from the Enterprise Data Warehouse and by chart review by study personnel (JG) using REDCap®. Data gathered from the Enterprise Data Warehouse included patient demographics (age at admission, gender, and self-reported ethnic group), hospital length of stay, intensive care unit (ICU) admission status, receipt of Infectious Diseases (ID) consultation, receipt of echocardiogram, positive blood culture with Streptococcal species, index vitals and labs, date of death, and date of readmission. Data gathered by manual chart review included chronic conditions present at the time of hospital admission, presence of indwelling lines, cardiac prosthetics or devices or other prosthetics, immunosuppression status (Table S1), history of intravenous drug use (IVDU), vitals and labs on day 3 from index blood culture, index absolute neutrophil count (ANC), repeat blood cultures, infection source, source control procedures, inpatient and outpatient antibiotics, duration of antibiotic therapy, and date of repeat BSI. The source of infection was determined from ID consultation documentation, if available, or primary team providers’ discharge documentation.

### Statistical analysis

To account for potential confounders in the decision to treat Streptococcal BSI with a short versus long antibiotic duration, we used inverse probability of treatment weighting (IPTW) to estimate the average treatment effects (ATE) of in vitro active antibiotics administered for 10 days or fewer versus more than 10 days, adjusting for pretreatment variables. Potential confounders were identified *a priori* and include source of infection, index ANC, ID consultation, chronic conditions, cardiac and other prosthetic devices, history of IVDU, index blood pressure and respiratory rate, receipt of immunosuppressive medication, age and admission to ICU (Figure S2).

**Figure 1.**
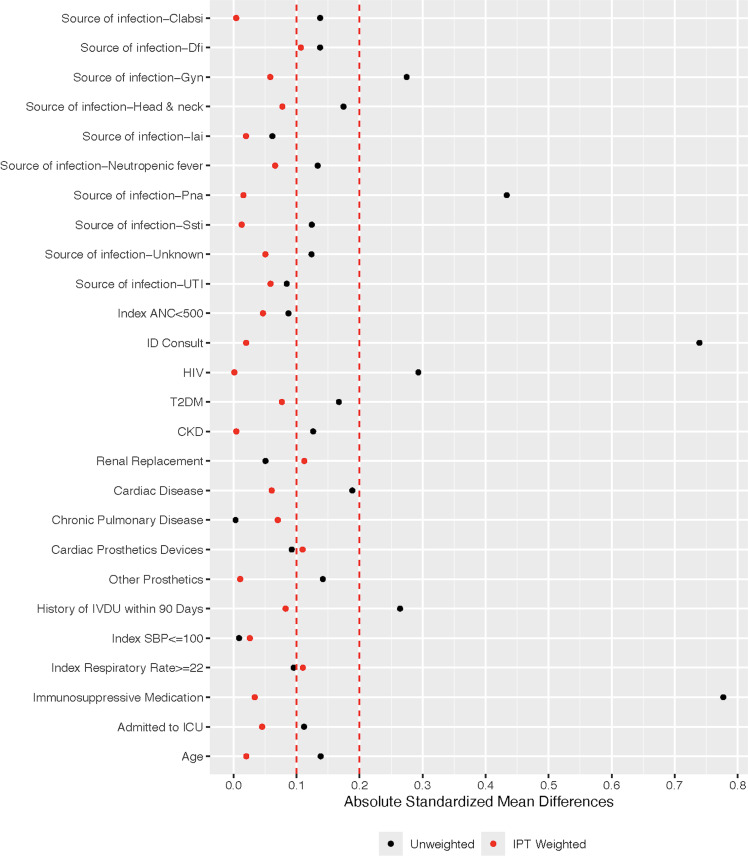
Absolute standardized mean difference.

We first assessed balance in these variables between the treatment groups using absolute standardized mean differences (ASMDs).^16,17^ Variables with the highest ASMDs (>0.2 or close to 0.2) were included in a multivariable logistic regression model to predict treatment assignment. The predicted probabilities (ie propensity scores) were then used to construct the IPT weights which were truncated at 99^th^ percentile to minimize the impact of individuals with disproportionately large weights. We then reassessed ASMDs of all variables between the weighted groups. If there were still variables with large ASMDs, we revised the propensity score model by including additional variables and removing correlated variables then recalculated propensity scores and IPT weights. This iterative process continued until all variables were adequately balanced between treatment groups, indicated by ideally, ASMDs smaller than or close to 0.1.^18^ We then examined the distributions of calculated propensity scores to ensure that the ranges of scores mostly overlapped between treatment groups (Figure 1).^18,19^ Additionally, we performed a separate sensitivity analysis comparing outcomes from index blood culture rather than from EOT.

With subjects weighted by IPTW, we estimated the ATE of short antibiotic duration using logistic regression for binary outcomes and gamma regression (a generalized linear model assuming a gamma distribution of the response variable given the predictors) for length of stay (LOS) because of its positive nature and right-skewed distribution. Odds ratios (or ratio for the LOS outcome) were reported with 95% confidence intervals (CIs) and P-values. Predicted outcomes by treatment groups were also reported. All analyses were conducted in R v4.3 and all tests were two sided. Total counts and percentages, median and interquartile ranges, and means and standard deviations were used to summarize data as appropriate.

## Results

**Figure 2.**
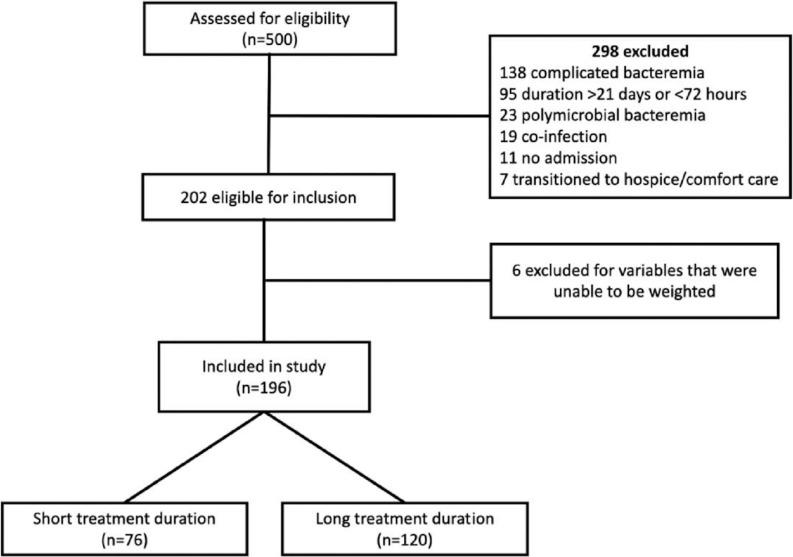
Consort diagram.

Five-hundred unique patient encounters were screened (Figure 2) and 202 met criteria for inclusion. The most common reason for exclusion was due to complicated BSI (Figure 2). Six patients were later excluded for having predicted probability of 0 or 1 for receiving either treatment. Among the remaining 196 patients, 76 were in the short treatment group and 120 in the long treatment group. Propensity scores had satisfactory overlap between the short and long-treatment groups (Figure 1) and all potential confounders were adequately balanced after IPT weighting.

Skin and soft tissue infections were the most common source of infection (35%), followed by pneumonia (28%), and intra-abdominal infections (12%). The most common *Streptococcus* species isolated were Viridans Group (30.1%), *S. pyogenes* (27.6%), and *S. pneumoniae* (19.4%). One-third of patients were admitted to the ICU (32%) and 30% had a cardiac or other prosthetic device. There were more patients with an ID consultation in the long treatment group compared to the short treatment group (Table 1). The median durations of therapy were 8 (IQR, 7–10) and 15 (IQR, 14–17) days in the short and long treatment groups, respectively.

**Table 1.** Baseline characteristics

Variable	Total (N = 196)	Short (N = 76)	Long (N = 120)
Age, Mean (SD)	57 (17)	58 (18)	56 (16)
Sex			
Male	105 (53.6%)	41 (53.9%)	64 (53.3%)
Female	91 (46.4%)	35 (46.1%)	56 (46.7%)
Source of Infection			
SSTI	69 (35%)	24 (32%)	45 (38%)
Pneumonia	54 (28%)	30 (39%)	24 (20%)
Intra-abdominal	23 (12%)	8 (11%)	15 (12%)
Unknown	17 (8.7%)	5 (6.6%)	12 (10%)
Gynecologic	9 (4.6%)	1 (1.3%)	8 (6.7%)
UTI	7 (3.6%)	2 (2.6%)	5 (4.2%)
Head & neck	6 (3.1%)	1 (1.3%)	5 (4.2%)
Neutropenic fever	5 (2.6%)	1 (1.3%)	4 (3.3%)
CLABSI	3 (1.5%)	2 (2.6%)	1 (0.83%)
Diabetic Foot Infection	3 (1.5%)	2 (2.6%)	1 (0.83%)
Infectious Diseases Consultation	73 (37%)	13 (17%)	60 (50%)
HIV	8 (4.1%)	6 (7.9%)	2 (1.7%)
Type II Diabetes	48 (24%)	22 (29%)	26 (22%)
CKD	21 (11%)	10 (13%)	11 (9.2%)
Renal Replacement	9 (4.6%)	3 (3.9%)	6 (5%)
Cardiac Disease	97 (49%)	42 (55%)	55 (46%)
Chronic Pulmonary Disease	44 (22%)	17 (22%)	27 (22%)
Cardiac Prosthetic Devices^a^	27 (14%)	9 (12%)	18 (15%)
Other Prosthetics^b^	32 (16%)	10 (13%)	22 (18%)
History of IVDU within 90 Days	16 (8.2%)	3 (3.9%)	13 (11%)
Index SBP ≤ 100	28 (14%)	11 (14%)	17 (14%)
Index Respiratory Rate ≥ 22	82 (42%)	34 (45%)	48 (40%)
Index ANC <500	10 (5.1%)	3 (3.9%)	7 (5.8%)
Immunosuppressive Medication^c^	75 (38%)	13 (17%)	62 (52%)
Admitted to ICU	63 (32%)	22 (29%)	41 (34%)
Streptococcal Species			
Viridans Group^d^	59 (30.1%)	22 (28.9%)	37 (30.8%)
*S. pyogenes*	54 (27.6%)	13 (17.1%)	41 (34.2%)
*S. pneumoniae*	38 (19.4%)	27 (35.5%)	11 (9.2%)
*S. agalactiae*	22 (11.2%)	7 (9.2%)	15 (12.5%)
*S. dysgalactiae*	15 (7.7%)	6 (7.9%)	9 (7.5%)
*S. bovis group*	8 (4.1%)	1 (1.3%)	7 (5.8%)

Abbreviations: ANC, absolute neutrophil count; CLABSI, central line associated bloodstream infection; CKD, chronic kidney disease; HIV, human immunodeficiency virus; IVDU, intravenous drug use; SBP, systolic blood pressure; SD, standard deviation; SSTI, skin and soft tissue infection; UTI, urinary tract infection.

a Cardiac prosthetics include implantable cardiac electronic devices, including pacemakers, implantable cardiac defibrillators and cardiac resynchronization therapy devices, vascular grafts, prosthetic & bioprosthetic valves, ventriculoatrial shunts, and ventricular assist devices.

b Other prosthetics include active dialysis graft, orthopedic fixation devices, or orthopedic replacement hardware.

c History of systemic chemotherapy within previous 6 weeks of index admission, Receipt of high dose steroids (>20 mg per day of prednisone equivalent) for >2 weeks, or any history of the following immunosuppressive medication in prior 3 months of index admission: abatacept, adalimumab, azathioprine, brodalumab, certolizumab, cyclosporine (systemic), etanercept, everolimus, golimumab, infliximab, ixekizumab, mercaptopurine, methotrexate (>20 mg/wk), mycophenolate, rituximab, sirolilmus, secukinumab, tacrolimus, tocilizumab, tofacitinib, ustekinumab.

d Viridans Group Streptococci as reported in the electronic health record: Viridans Group (37.3%), *S. mitis* group (30.5%), *S. anginosus* (23.7%), and *S. salivarius* group (8.5%).

Thirty-six patients (18%) met the primary composite outcome within 30 days from EOT. There was no statistically significant difference between short and long treatment durations in the odds of experiencing the primary outcome (OR 1.42; [95% CI: 0.57, 3.53]) or any of the secondary outcomes that were assessed within 90 days from the EOT, including death, repeat BSI, and readmissions, or hospital length of stay (Table 2). In the sensitivity analysis of the primary outcome, we found no difference between the short (18.0%) and long (12.0%) groups (OR 2.14; [95% CI: 0.82, 5.58]). Similarly, there were no significant differences in the sensitivity analysis of the secondary outcomes, including death (9.2% vs 4.2%; OR 3.00 [95% CI: 0.70, 12.88]), repeat BSI (2.6% vs 1.7%; OR 6.18; [95% CI: 0.65, 59.26]), and readmissions 26% vs 26%; OR 1.79; [0.81, 4.00]) for the short versus long groups.

**Table 2.** Primary and secondary outcomes

Outcome	Total (N = 203)	Short (N = 78)	Long (N = 125)	Odds ratio^a^ (95% CI)	P^a^-Value
Composite Outcome within 30 D from EOT	36 (18%)	14 (18%)	22 (18%)	1.42 (0.57,3.53)	0.45
Death within 30 D	3 (1.5%)	3 (3.9%)	0 (0%)		
Repeat BSI within 30 D	2 (1.0%)	1 (1.3%)	2 (0.83%)		
Readmission within 30 D	32 (16%)	11 (14%)	21 (18%)		
Death within 90 d from EOT	13 (6.6%)	7 (9.2%)	6 (5%)	2.60 (0.64, 10.59)	0.18
Repeat BSI within 90 D from EOT	4 (2%)	2 (2.6%)	2 (1.7%)	6.18 (0.65, 59.26)	0.11
Readmission within 90 D from EOT	53 (27%)	20 (26%)	33 (28%)	1.61 (0.73, 3.57)	0.24
LOS, Mean (SD)	7.3 (5.6)	7.0 (5.7)	7.5 (5.4)	1.03 (0.82, 1.30)	0.80

Abbreviations: EOT, end of treatment; LOS, length of stay.

a Effects of exposure (Short vs Long) on outcomes after IPT weighting.

## Discussion

Using an IPTW cohort study design, we found no difference in outcomes for patients treated with a short treatment duration (≤ 10 d) compared to a long treatment (> 10 d) duration for uncomplicated Streptococcal BSI. The incidence of all-cause mortality, repeat BSI, and unplanned readmissions at 30 days and 90 days from EOT was similar between the two groups and there was no significant difference for hospital length of stay. Additionally, there were no differences for any primary or secondary outcomes in the sensitivity analyses from date of index blood culture. These results add to the growing body of literature demonstrating the safety and effectiveness of using shorter treatment durations for many infectious diseases and challenges the current standard practice of a 14-day treatment course for uncomplicated Streptococcal BSI.

There has been a push to reconsider the treatment durations for many infectious diseases including pneumonia, urinary tract infections, intra-abdominal infections, and Gram-negative BSI. Yahav, et al. found no significant difference in BSI relapse, all-cause mortality, readmissions, or infectious complications comparing seven days of antibiotic treatment to 14 days for uncomplicated Gram-negative BSI in a prospective randomized non-inferiority study.^3^ This data has led to antimicrobial stewardship interventions at many institutions to shorten antibiotic durations for Gram-negative BSI and recognition of the lack of data to support current practice patterns for Gram-positive BSI.

In a retrospective cohort study of 286 BSI episodes, Nguyen, et al. compared outcomes for patients treated with short treatment duration (≤10 d) compared to long treatment duration (>10 d) for GAS BSI and found no difference in 90-day all-cause mortality or readmissions between the groups.^11^ In this study, patients with complicated and uncomplicated disease were included. Complicated disease was defined as the presence of empyema, myositis, septic arthritis, osteomyelitis, necrotizing fasciitis, infective endocarditis, or required surgical intervention or ICU admission. More patients with complicated disease were in the long treatment duration group, which may confound the evaluated outcomes of all-cause mortality at 30 and 90 days from admission and re-hospitalization within 30 days of discharge.^11^

Boulos, et al. published an abstract of a retrospective study comparing short-course therapy (≤10 d) to prolonged therapy (11–21 d) for uncomplicated Streptococcal BSI, excluding *S. pneumoniae*. The median durations of treatment were 8 and 15 days in the short and long-course groups. They found no significant differences between the short and long-treatment groups in recurrent BSI, readmissions, and all-cause mortality at 30 days from EOT.^12^ After completion of our analysis, additional retrospective evidence published by Clutter, et al found non-inferiority of a 5-to-10-day antibiotic duration compared to 11 to 15 days.^13^

There are limitations to consider regarding our findings, such as this was a retrospective cohort study with potential for residual confounding. We attempted to reduce the impact of bias through propensity score weighting, although short of a prospective randomized control trial, confounding is likely to persist. Our data also relied on manual data collection, which is limited by available documentation in the electronic health record. Additionally, it is possible patients with recurrent BSI presented to a hospital outside our facility and were not captured with manual chart review; however, we suspect both treatment groups would be equally susceptible to that missing data. Lastly, we observed low absolute rates of mortality and recurrent BSI limiting power to detect a meaningful difference in outcomes related to treatment duration. Conversely, low recurrent BSI rates in both groups highlight how infrequent proximal infection recurrence is that would potentially lead to worse outcomes, and we believe this low event rate overall should factor into treatment decisions where it is possible antibiotic-related adverse events could occur at a higher rate. Finally, we did not evaluate the impact of drug choice or dose on outcomes, specifically when patients are stepped down to oral therapy for definitive treatment. Although we are not aware of any data suggesting antibiotic durations need to be extended when using antibiotics with lower oral bioavailability. Our analysis includes several strengths, notably the employment of an IPTW strategy to mitigate the confounding inherent in observational study designs. Additionally, our study population includes patients with cardiac and other prosthetic devices, as well as those who received immunosuppressive medications and may be classified as immunosuppressed. These patient populations are classically considered to be at high risk of recurrent bacteremia and poor outcomes. Therefore, including them increases the generalizability of our findings to a broader range of patients.

In conclusion, we found no appreciable difference in outcomes comparing patients treated with ≤10 days of antibiotics compared to those treated with >10 days of antibiotics for uncomplicated Streptococcal BSI. Considering low absolute rates of recurrent BSI, coupled with lack of evidence of significant difference related to treatment duration, we believe it is reasonable to consider shorter treatment durations. Given limitations of this retrospective analysis and limited power, future investigation is warranted to validate our results; however, our data may serve as a basis for antimicrobial stewardship initiatives aimed at reducing treatment durations for uncomplicated Streptococcal BSI.
